# Type-1 Gall Bladder Perforation: Rare Complication of Cholelithiasis

**DOI:** 10.4103/1319-3767.74439

**Published:** 2011

**Authors:** Vipul D. Yagnik

**Affiliations:** Ronak Endo-Laparoscopy and General Surgical Hospital, Patan -384265, Gujarat, India. E-mail: vipul.yagnik@gmail.com

**Figure 1:**
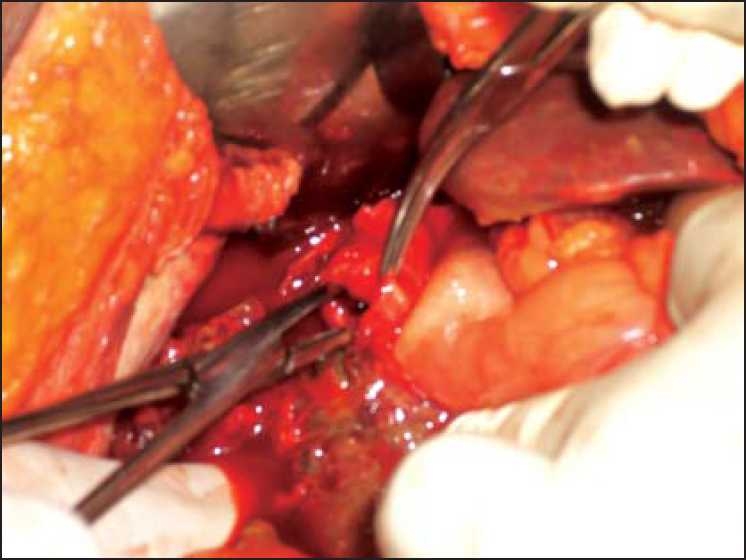
Perforation of the fundus of gall bladder

Sir,

A 45-year-old female presented with a history of abdominal pain, distention, and bilious vomiting since last 1 week. She had a history of fever and constipation since last 5 days. She denied any history of jaundice, smoking, and alcoholism. On examination she was dehydrated and tachycardic with low blood pressure. Her abdomen was distended and revealed signs of peritonitis. An erect chest radiograph showed pneumoperitoneum and ultrasound abdomen showed gross free fluid with internal echoes. After resuscitation with intravenous fluids, an exploratory laparotomy was performed. On opening the abdomen, frank purulent fluid was found. On exploration of the hepatobiliary region, a big perforation was seen over the fundus of the gall bladder with gallstone within [Figure 1]. Because of dense adhesion, it was not possible for us to remove the whole gall bladder, and thus we opted for a partial cholecystectomy. In the post-operative period, the patient developed minor bile leak which was treated by conservative measures.

Gall bladder perforation is an uncommon but life-threatening complication. Neimeier[1] classified gall bladder perforation in three categories (1) Type-1 (acute): free perforation with generalized peritonitis; (2) Type 2 (sub-acute): localized peritonitis; (3) Type 3 (chronic): cholecystoenteric fistula. Most of the cases of gall bladder perforation can only be diagnosed during surgery.[2] Free perforation (<1%) is less common and occurs only if greater omentum is unable to cover the inflammatory condition. In most of the patients, impacted stone in the cystic duct slips back in the gallbladder and enables the cholecystitis to resolve. If cholecystitis does not resolve due to persistent impaction of stone, inflammation may progress, an empyema may develop. Persistent inflammation and gallbladder distension due to impacted stone leads to ischemia, necrosis, and perforation. These types of patients require aggressive fluid ressuciatation, anti-microbial, and emergency exploratory laparotomy. The mortality rate is between 19 and 24%.[3]
